# Hydrology in the 21st century: challenges in science, to policy and practice

**DOI:** 10.1098/rsta.2024.0299

**Published:** 2025-07-31

**Authors:** Hayley J. Fowler, Gemma Coxon, Christopher J. White

**Affiliations:** ^1^ School of Engineering and Tyndall Centre for Climate Change Research, Newcastle University, Newcastle upon Tyne, UK; ^2^ School of Geographical Sciences, University of Bristol, Bristol, UK; ^3^ Department of Civil and Environmental Engineering, University of Strathclyde, Glasgow, UK

**Keywords:** forecasting, climate, policy

## Abstract

To mark the 40th anniversary of the British Hydrological Society, a landmark Discussion Meeting was held at the Royal Society in June 2024, bringing together a transdisciplinary community, including hydrologists, policymakers and practitioners, to reflect on four decades of progress and chart future directions for hydrology. This special issue presents a collection of papers from that meeting, addressing advances in data, modelling, forecasting and decision-making in the context of intensifying climate and hydrological extremes. Key themes include the need for open, reproducible science, greater integration of machine learning, AI and convection-permitting models and a shift towards transdisciplinary, co-produced knowledge that better supports adaptation, resilience and policy impact. The issue highlights the critical roles of education, collaboration and equity in shaping a hydrology that is not only technically advanced but socially and environmentally responsive to the challenges of the 21st century.

This article is part of the Royal Society Science+ meeting issue ‘Hydrology in the 21st century: challenges in science, to policy and practice’.

## 1. Introduction

The first meeting of the newly established British Hydrological Society (BHS) took place at the Royal Society in November 1983, following its formation at a 1982 International Association of Hydrological Sciences meeting in Exeter. To mark its 40th anniversary, a Discussion Meeting was held at the Royal Society in July 2024, bringing together researchers, practitioners and policymakers to reflect on how far hydrology has progressed and where it must go next. This special issue presents papers from that meeting, which aimed to synthesize advances in hydrological science over the past four decades, identify current challenges and explore opportunities for future progress.

Key objectives included: (1) examining state-of-the-art methods, models, data and observations for tackling hydrological extremes and broader challenges such as climate change; (2) highlighting the value of transdisciplinary collaboration, and (3) identifying ways to accelerate the uptake of new science into policy and practice. The meeting provided a timely platform to assess progress, benchmark current capabilities and consider how hydrology—when combined with expertise from other disciplines—can support more effective responses to today’s pressing societal and environmental challenges.

The meeting brought together hydrologists from around the world alongside the UK hydrological community to explore the future of hydrology and its role in addressing major societal challenges such as climate change. While the rate at which hydrological extremes are changing remains uncertain, their frequency and intensity appear to be increasing (e.g. [1]). Effectively managing and adapting to these emerging extremes requires a deeper understanding of the full cascade of processes—from precipitation and catchment hydrology [2,3] to downstream impacts on infrastructure and ecosystems (e.g. [4]).

In addition to the effects of global warming (e.g. [5]), hydrological systems are under pressure from population growth and increased resource use. Meeting these challenges demands new datasets, methods and models that are open-source, quality-controlled and verified—enabling collaborative, cross-disciplinary knowledge creation (e.g. [6]). Such transdisciplinary co-creation is essential for developing timely, actionable insights that can be translated into policy and practice, supporting informed decision-making and societal transformation in the face of the climate crisis.

The Discussion Meeting was structured around four core themes that examined progress over the past 40 years, identified persistent challenges and explored opportunities for future innovation: (i) challenges in data and observations, (ii) challenges in methods and modelling, (iii) managing hydrological extremes and grand challenges, and (iv) future perspectives for progress. Each theme featured a series of talks and discussions that brought together hydrologists, interdisciplinary scientists and leaders from industry, policy and decision-making.

The sessions showcased groundbreaking developments including open-source data and models, emerging sensor technologies, advanced modelling techniques, early warning systems and applications of machine learning and AI. Broader topics such as climate change adaptation, risk communication and decision-making were also explored, linking scientific innovation to real-world impact. Panel discussions fostered cross-sector dialogue, helping to shape new directions for managing hydrological extremes and enhancing societal resilience in an increasingly uncertain climate.

### (a) Challenges to hydrology in the 21st century

This special issue starts with Hall *et al.* [7], who critically examine the growing disconnect between hydrological science and the complex evidence needs of decision-makers managing water resources in an era of climate change. They argue that despite past successes in risk-based modelling and forecasting, current hydrological science lacks the modelling fidelity, spatial resolution and integration needed to guide decisions on flooding, droughts, water quality and ecosystem resilience. The authors call for advances in physically based, high-resolution modelling of catchments that can simulate the aggregate impact of multiple interventions (e.g. natural flood management, urban drainage, groundwater abstraction), as well as their performance under uncertain and extreme future conditions. They emphasize the importance of coupling hydrology with socio-economic modelling to capture human–water interactions, and advocate for the integration of simulation tools with modern decision-support frameworks like adaptive pathways and robust decision-making. Overall, the paper argues that to support sustainable and adaptive water governance, hydrology must evolve into a more interdisciplinary, data-rich and decision-driven science.

In the second paper, Davies *et al.* [8] outlines the historical evolution and future trajectory of operational meteorology, arguing for a shift towards transdisciplinary collaboration to improve societal resilience to weather and climate extremes. Tracing a lineage from Admiral FitzRoy’s 19th-century gale warnings through WWII’s D-Day forecasts and the 1987 Great Storm, the authors show how meteorology has evolved from a single-discipline focus to more collaborative, impact-based systems. Milestones include the development of the UK’s National Severe Weather Warning Service and Flood Forecasting Centre, which integrated meteorology and hydrology into a new hydrometeorological profession. Looking ahead, the paper advocates for blending machine learning, ensemble forecasting, causal inference and storyline methods with behavioural and decision sciences to support complex, actionable decision-making. Tools like digital twins and virtual/augmented reality are highlighted for their potential to enhance public comprehension and preparedness. The authors emphasize that effective services require trust, co-design with users and sustained collaboration across government, business, academia and communities—especially through living labs. They conclude that the operational meteorologist of the future will be not just a forecaster, but a climate science translator, socio-meteorologist and trusted advisor enabling whole-of-society responses to extreme weather.

Next, Orr *et al.* [9] argue that hydrological research must evolve to better support climate adaptation and societal resilience. Authored by a team spanning academia, government and policy, it emphasizes that while the science of hydrology is advancing, its influence on real-world decisions remains limited by institutional silos, poor communication and misalignment with policy and community needs. The authors advocate for transdisciplinary, participatory research that co-produces knowledge with decision-makers, communities and industry partners. They present practical case studies—from climate flood allowances to riparian shading projects—that illustrate how co-created, context-sensitive solutions can lead to better outcomes. The paper also calls for systemic change in how impact is defined and rewarded, urging a shift from citation-based metrics to those recognizing engagement, relevance and resilience-building. Ultimately, it makes a strong case that the hydrology community must prioritize relationship-building, equity and actionable knowledge to address the escalating challenges of climate change.

Speight [10] argues that effectively addressing climate-driven hydrological risks requires placing people—both as individuals and within society—at the heart of hydrological science, education, decision-making and practice. They show that despite significant scientific advances, hydrology often fails to influence real-world outcomes because of gaps in communication, public engagement and interdisciplinary integration. The author explores how challenges such as complacency, limited capacity, complex information and poor communication hinder action, especially in the context of flood warnings. She highlights the need for hydrologists to go beyond technical expertise and adopt roles as communicators, collaborators and systems thinkers. The paper emphasizes the importance of rethinking hydrology education to better reflect human–water interactions, supporting interdisciplinary training and embedding social science perspectives. Ultimately, the opinion piece calls for a more people-centred hydrology that builds trust, improves decision-making and ensures that hydrological knowledge translates into meaningful climate adaptation and risk reduction.

### (b) The need for new open-source datasets and improved methods

We next present a series of papers that examine the need for advances in datasets and methods used for hydrological analysis and implementation of the science.

We start with Slater *et al.* [11] who explore the transformative potential of machine learning (ML) and explainable AI (XAI) in hydrology, highlighting their success in outperforming traditional models for simulation, forecasting and data generation. They demonstrate how ML models, especially deep learning architectures like long short-term memory (LSTM) models, can reveal new hydrological insights when paired with XAI techniques such as SHapley Additive ExPlanations (SHAP), partial dependence plots (PDPs) and gradient-based methods. The authors emphasize that while ML enables powerful predictions and process discovery, challenges remain around model interpretability, causal inference, uncertainty quantification and prediction in data-scarce or human-altered regions. They advocate for a cautious and transparent application of XAI, viewing it as a hypothesis-generating tool rather than a definitive explanation of physical processes. The paper concludes by encouraging interdisciplinary collaboration, openness about failures and development of multivariate and physics-informed models to fully harness the benefits of ML in a data-rich but uncertain hydrological future.

This is followed by Nathan *et al.* [12] who critique the widespread use of deterministic, event-based flood models that assume the exceedance probability of a flood matches that of the triggering rainfall. The paper argues that this assumption is fundamentally flawed—especially under a changing climate—and advocates for a shift to stochastic modelling frameworks that explicitly account for aleatory uncertainty (natural variability in rainfall, soil moisture and storm profiles) and epistemic uncertainty (knowledge gaps, data limitations, model choice). Using a case study from the Delatite River catchment in Australia, the author demonstrates how traditional methods can be misleading and how simple stochastic adaptations—using Monte Carlo simulation—can greatly improve the realism and defensibility of flood frequency estimates. The paper highlights that event-based models, due to their simplicity and adaptability, are particularly well-suited for incorporating climate change impacts, such as increases in rainfall intensity and changes in soil moisture or storm structure. They emphasize the disconnect between academic flood science and the practical tools used by industry and call for a targeted research agenda to develop and regionalize stochastic event-based tools that bridge this gap.

Dale [13] discusses the crucial role of convection-permitting models (CPMs) in managing hydrological extremes such as flash floods and sewer overflows. CPMs are advanced weather and climate models capable of simulating intense, localized convective rainfall with high spatial and temporal resolution—something traditional models struggle to achieve. The paper presents two real-world case studies: (i) a prototype flood alert system in Freetown, Sierra Leone, co-developed with local agencies using ensemble CPM forecasts to provide early warning of high-impact convective storms and (ii) the RED-UP tool in the UK, which perturbs historical rainfall time series based on CPM climate projections to help water companies assess future sewer overflow risks under climate change. Both cases underscore the benefits of CPMs in both short-term forecasting and long-term planning, demonstrating their practical value when designed in collaboration with end users. The paper concludes by highlighting future opportunities, including the integration of AI with CPMs, the importance of ensemble and probabilistic approaches and the need for international collaboration to improve data and modelling capacity—particularly in data-scarce regions like West Africa.

### (c) The need for education, open and reproducible research and transdisciplinary frameworks

The final three papers provide thoughtful perspectives on how education, reproducible research and transdisciplinary frameworks might be used to advance hydrological research through policy and practice.

First, Selker [14] describes the transformative model of the Openly Published Environmental Sensing (OPEnS) Lab at Oregon State University. Founded in 2015, OPEnS is an undergraduate-led engineering lab that develops open-source environmental sensor technologies addressing real-world challenges in hydrology, climate, agriculture and ecology. Combining cutting-edge tools—like micro-electromechanical systems (MEMS) sensors, three-dimensional printing, microcontrollers, telemetry and cloud platforms—with a hands-on, student-centred educational philosophy, OPEnS empowers students to design, build and deploy sensing systems that are ‘Born FAIR’ (findable, accessible, interoperable, reusable). The lab has yielded over 28 innovative projects, 14 peer-reviewed papers, patents and collaborations with scientists, agencies and industries worldwide. Notable systems include eDNA water samplers, high-precision dendrometers and fire-resilient air quality monitors. The paper emphasizes that engaging students in value-driven, interdisciplinary, problem-solving projects produces not only better sensing tools but also highly skilled graduates. Selker advocates scaling the OPEnS model through international ‘sister labs’ and calls for community-wide efforts to adopt interoperable sensor metadata standards and democratize environmental data. The lab demonstrates how connecting students, scientists and industry can accelerate both research and innovation in hydrology.

Hut & Hall [15] propose a transformative approach to improve reproducibility, accessibility and equity in hydrological research. They introduce the MRDTACS principle: research should be reproducible by individuals earlier in their academic careers—and in less time—than it took the original researchers. The authors argue that embedding Open and FAIR practices, using reproducibility-friendly platforms (e.g. eWaterCycle, HydroShare, Google Colab) and teaching dependency management, containerization and modular workflows are essential to this goal. The paper provides practical strategies and tools to help early-career researchers and students reproduce and extend existing work efficiently. Through a case study on the eWaterCycle platform, the authors illustrate how technical design choices can reduce barriers, foster collaboration and improve education. Ultimately, they advocate for a cultural shift in hydrology where reproducibility and inclusivity are prioritized to accelerate innovation and empower the next generation of scientists.

Finally, Hall & Melvold [7] argue that to address today’s complex and climate-driven water challenges, hydrology must evolve from a technically focused science to one that is embedded in social, political and cultural contexts. Traditional hydrology often neglects these broader dimensions, limiting its real-world impact. The authors advocate for transdisciplinary approaches, which integrate diverse knowledge systems—including local, indigenous and stakeholder perspectives—through co-production and collaborative problem-solving. They introduce and evaluate three established transdisciplinary frameworks (The Ten Essentials, Integration and Implementation Sciences (i2S) and the ISOE model), each offering guidance on reflexivity, uncertainty management and stakeholder engagement. The paper proposes four core principles—knowledge translation, diverse knowledge integration, adaptive methodologies and reflexivity—and offers five guiding questions to help hydrologists align their work with societal needs. Ultimately, the authors call for a shift in hydrological research culture that embraces transdisciplinary methods to generate more ethical, inclusive and actionable solutions in the face of global water crises.

## 2. Overarching themes and future directions

The papers in this special issue collectively chart a bold vision for the future of hydrology—one that is more open, inclusive and impact-oriented. While the field has made significant advances over the past four decades, the challenges of climate change, increasing hydrological extremes and complex socio-environmental pressures demand a step change in how hydrological science is conducted, communicated and applied.

Across the contributions, several unifying themes emerge: the urgent need for transdisciplinary collaboration, the importance of open and reproducible science, the centrality of climate adaptation and the role of education and equity in shaping the next generation of hydrologists. Together, these themes point towards a future in which hydrology is not only a technical discipline but also a key enabler of societal resilience and transformation. To highlight the priority directions for research, practice and policy:

(1) *Transdisciplinary collaboration.* Hydrology must move beyond technical silos to engage with social sciences, policy, communities and industry through co-production and mutual learning.
(2) *Open, accessible and actionable science.* There is a pressing need for open-source data, reproducible tools and quality-controlled models that can be quickly translated into policy and practice.
(3) *Climate change and hydrological extremes.* Understanding and adapting to the intensification of hydrological extremes requires advanced modelling, better forecasting and scenario-based planning under uncertainty.
(4) *Education and capacity building*. Training the next generation of hydrologists must involve interdisciplinary problem-solving, hands-on projects and a strong foundation in reproducibility and communication.
(5) *Cultural shift in research evaluation*. The hydrology community must redefine ‘impact’ to value engagement, inclusion and resilience-building—moving beyond citations towards meaningful change.

This special issue sets a clear agenda: hydrology must become more open, transdisciplinary and socially relevant to meet the urgent demands of a rapidly changing world.

## Data Availability

This article has no additional data.

## References

[1] Fowler HJ *et al*. 2021 Anthropogenic intensification of short-duration rainfall extremes. Nat. Rev. Earth Environ. **2**, 107–122. (10.1038/s43017-020-00128-6)

[2] Beven K *et al*. 2020 Commentary: developing observational methods to drive future hydrological science: can we make a start as a community? Hydrol. Process. **34**, 868–873. (10.1002/hyp.13622)

[3] Wagener T *et al*. 2022 Knowledge gaps in our perceptual model of Great Britain’s hydrology. Hydrol. Process. **35**, e14288. (10.1002/hyp.14288)

[4] Orr HG, Ekström M, Charlton MB, Peat KL, Fowler HJ. 2021 Using high-resolution climate change information in water management: a decision-makers’ perspective. Phil. Trans. R. Soc. A. **379**, 20200219. (10.1098/rsta.2020.0219) 33641469

[5] Vosper E, Betts R, Challinor A, Fowler HJ, Mitchell D. 2019 Building a UK climate impacts and risk assessment community. Weather **74**, 307–309. (10.1002/wea.3592)

[6] Lamb R *et al*. 2022 The future of flood hydrology in the UK. Hydrol. Res. **53**, 1286–1303. (10.2166/nh.2022.053)

[7] Hall C, Melvold J. 2025 Advancing hydrology for societal impact: integrating transdisciplinary frameworks to bridge research and practice. Phil. Trans. R. Soc. A **383**, 20240283. (10.1098/rsta.2024.0283) 40739921 PMC12311488

[8] Davies P, Fowler HJ, Roberts H, White CJ, Youngman M, Rogers DP. 2025 The changing role of operational meteorology towards a transdisciplinary approach to future weather and climate services. Phil. Trans. R. Soc. A **383**, 20240535. (10.1098/rsta.2024.0535) 40739915 PMC12311484

[9] Orr HG, Hall CA, Rhodes V, Wilby RL, Peat KL, Fowler HJ. 2025 Hydrology for impact: building partnerships, blending knowledge and bracing for climate change. Phil. Trans. R. Soc. A **383**, 20240290. (10.1098/rsta.2024.0290) 40739925 PMC12311487

[10] Speight L. 2025 Why people matter in 21st century hydrology: lessons learnt from flood forecasting and warning. Phil. Trans. R. Soc. A **383**, 20240293. (10.1098/rsta.2024.0293) 40739922 PMC12311491

[11] Slater L *et al*. 2025 Challenges and opportunities of ML and explainable AI in large-sample hydrology. Phil. Trans. R. Soc. A **383**, 20240287. (10.1098/rsta.2024.0287) 40739919 PMC12334205

[12] Nathan R. 2025 Improving event-based methods for modelling flood risk in a variable and non-stationary climate. Phil. Trans. R. Soc. A **383**, 20240292. (10.1098/rsta.2024.0292) 40739923 PMC12311489

[13] Dale M, Shelton K. 2025 Convection-permitting models for managing hydrological extremes: practical, innovative examples. Phil. Trans. R. Soc. A **383**, 20240289. (10.1098/rsta.2024.0289) 40739916

[14] Selker J, Udell C. 2025 Education and technology synergy in environmental sensing. Phil. Trans. R. Soc. A **383**, 20240281. (10.1098/rsta.2024.0281) 40739924 PMC12311486

[15] Hut R, Hall C. 2025 More efficient reproducible research in hydrology: moving research down the academic career scale (MRDTACS). Phil. Trans. R. Soc. A **383**, 20240296. (10.1098/rsta.2024.0296) 40739920 PMC12311485

